# UMUD: a web application for easy access to musculoskeletal ultrasonography datasets

**DOI:** 10.1186/s12880-026-02170-0

**Published:** 2026-02-06

**Authors:** Paul Ritsche, Fabio Sarto, Francesco Santini, Christoph Leitner, Martino V. Franchi, Oliver Faude, Taija Finni, Olivier Seynnes, Neil Cronin

**Affiliations:** 1 Department of Sport, Exercise and Health, University of Basel, Basel, Switzerland; 2 Department of Biomedical Sciences, University of Padova, Padova, Italy; 3 Basel Muscle MRI, Department of Biomedical Engineering, University of Basel, Basel, Switzerland; 4 Department of Radiology, University Hospital of Basel, Basel, Switzerland; 5 Department of Information Technology and Electrical Engineering, ETH Zurich, Zurich, Switzerland; 6 Faculty of Sport and Health Sciences, University of Jyvaskyla, Jyvaskyla, Finland; 7 Department for Physical Performance, Norwegian School of Sport Sciences, Oslo, Norway; 8 School of Education & Science, University of Gloucestershire, Gloucester, UK

**Keywords:** Benchmarks, Database, Data sharing, Muscle architecture, Muscle cross-sectional area, Open access, Open science, Public repositories, Tendon, Ultrasound imaging

## Abstract

**Background:**

Ultrasonography is widely used to assess skeletal muscle and tendon properties, such as architecture, cross-sectional area, and tissue stiffness. Despite its growing application in different scenarios, the lack of accessible and standardized public datasets limits large-scale studies and the development of image analysis algorithms. To address this, we developed the Universal Musculoskeletal Ultrasonography Database (UMUD), a platform designed to facilitate access to these datasets and foster standardization in musculoskeletal ultrasonography imaging research. UMUD is an online repository that aggregates and indexes metadata from publicly available musculoskeletal ultrasonography datasets hosted on platforms like the Open Science Framework and Zenodo. By offering detailed metadata descriptors—such as muscle group, ultrasound device, participant demographics, and publication details—UMUD streamlines dataset discovery and exploration through search and visualization tools.

**Results:**

So far, UMUD includes 14 Datasets, including 76.124 images (and/or 2.674 videos) derived from 1.901 participants. The platform also includes benchmark datasets for training and validating image analysis algorithms. These comprise multi-expert analyses of muscle architecture and panoramic cross-sectional area images, a dataset containing overlays of muscle geometry for teaching muscle architecture analysis, and labeled datasets for training deep learning models. Additionally, UMUD lists state-of-the-art automated analysis algorithms to support users in their application.

**Conclusion:**

UMUD addresses relevant challenges in musculoskeletal ultrasonography by providing a centralized, standardized repository of datasets and tools. Thus, it promotes transparency and innovation in the field, supporting reproducible research and advancements in automated image analysis. Future developments include adding more datasets, refining user functionalities, and introducing community-driven challenges to enhance its impact.

**Supplementary Information:**

The online version contains supplementary material available at 10.1186/s12880-026-02170-0.

## Background

Ultrasonography provides a non-invasive, non-ionizing, and cost-effective imaging modality with deep tissue penetration capabilities that has become instrumental in assessing key properties of musculoskeletal tissues, such as skeletal muscle architecture and anatomical cross-sectional area, tendon size, and stiffness [1, 2]. These parameters have been widely applied to study associations with muscle function and physical performance [3] and adaptations to sports practice and exercise training [1], aging and sarcopenia [4], muscle disuse [5], and musculoskeletal disorders [6]. Moreover, musculoskeletal ultrasound has recently been explored as a neurorobotic interface, leveraging its real-time operating capabilities to control human-computer interfaces, addressing trade-offs associated with other modalities [7]. Despite the growing recognition of ultrasonography’s value in musculoskeletal research, the developments in artificial intelligence applications in this field [8] and the increasing call for open data access and sharing in clinical research [9], there remains a significant scarcity of public datasets in this field. This issue poses challenges for researchers, as it limits opportunities to share, compare, and leverage existing data for large-scale studies and machine learning image analysis applications. This is particularly relevant given the challenges of manual analysis, which is operator-dependent and time-consuming [1]. Even when datasets are available, these are often not easily findable and reusable and lack consistency in imaging protocols, labeling conventions, and data formats (i.e [10]. vs. https://www.cs.cit.tum.de/camp/publications/leg-3d-us-dataset/, comparison made with FAIR-Checker [11]). Traditional repositories (e.g., The Open Science Framework or Zenodo) often provide inconsistent and domain-unspecific metadata, resulting in reduced findability and usability of datasets [11]. Without standardized and accessible databases, the musculoskeletal ultrasonography imaging community will continue struggling to establish common ground truths, standardized training datasets, and benchmark datasets for novel image analysis tools. This lack of shared resources not only hinders the development of robust image analysis algorithms employing deep neural networks—which may provide the most efficient and universal approach but requires very large datasets—but also limits opportunities to train researchers in best practices for analyzing and interpreting ultrasonography data.

The availability of centralized and curated databases has been transformative in other fields, as seen with platforms like Hugging Face [12], ImageNet [13, 14], and DataHub.io (https://datahub.io/). These resources have sparked significant advancements by enabling researchers’ widespread access to datasets, to test and refine algorithms, compare methodologies, and collaborate across disciplines [15]. Within the musculoskeletal imaging community, initiatives from the Open Repository for Musculoskeletal Imaging Research (ORMIR) [16] already foster innovation and enhance transparency and reproducibility in the field. Establishing community-driven repositories for ultrasonography imaging would further democratize access to high-quality datasets, promote standardization, and accelerate progress in musculoskeletal research. While open access data platforms exist for magnetic resonance images (i.e., http://mridata.org/, https://openneuro.org/) and radiological ultrasonography (i.e., https://qamebi.com/breast-ultrasound-images-database/), no curated, centralized, open-access database of musculoskeletal ultrasonography images in healthy or clinical populations is available.

**Fig. 1:**
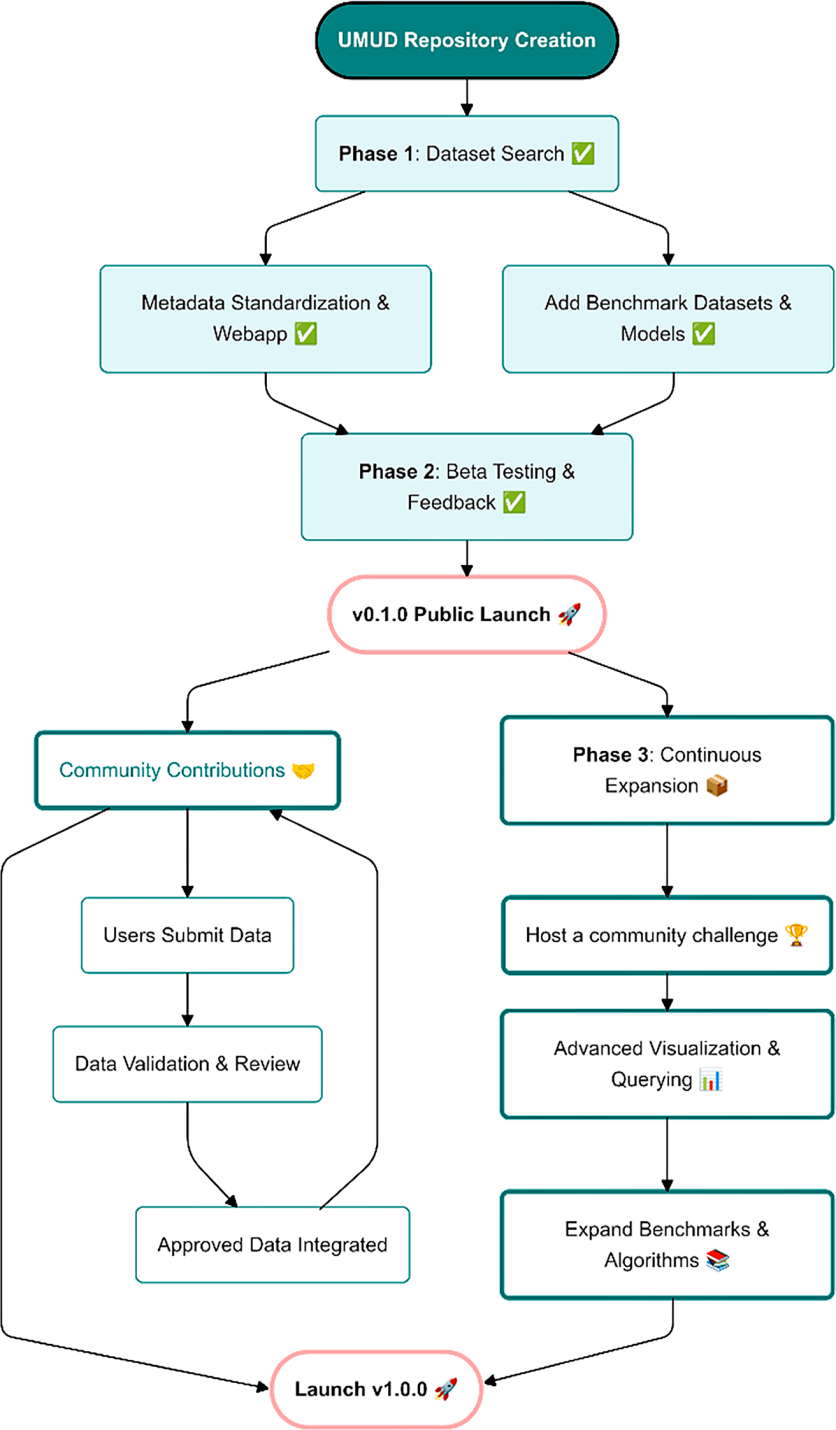
The UMUD roadmap, highlighting completed development steps and future directions

Here we introduce the Universal Musculoskeletal Ultrasonography Database (UMUD). This centralized platform available online (https://universalmuscledatabase.streamlit.app/) is designed to streamline the findability of musculoskeletal ultrasonography datasets using standardized metadata descriptors. UMUD aggregates metadata from various public datasets, including 2D B-mode images, videos, and 3D ultrasonography data (collected using 1D arrays and reconstructed). In coherence with the FAIR principles [17, 18], UMUD aims to facilitate the efficient discovery and use of datasets, easing their findability through comprehensive metadata indexing and user-friendly search tools. In addition, as part of the ORMIR community [16], UMUD also sets the stage for establishing analysis standards, benchmarking datasets, and fostering collaboration between researchers in the field (see Fig. 1 for an overview of completed and future development steps).

## Construction and content

**Fig. 2:**
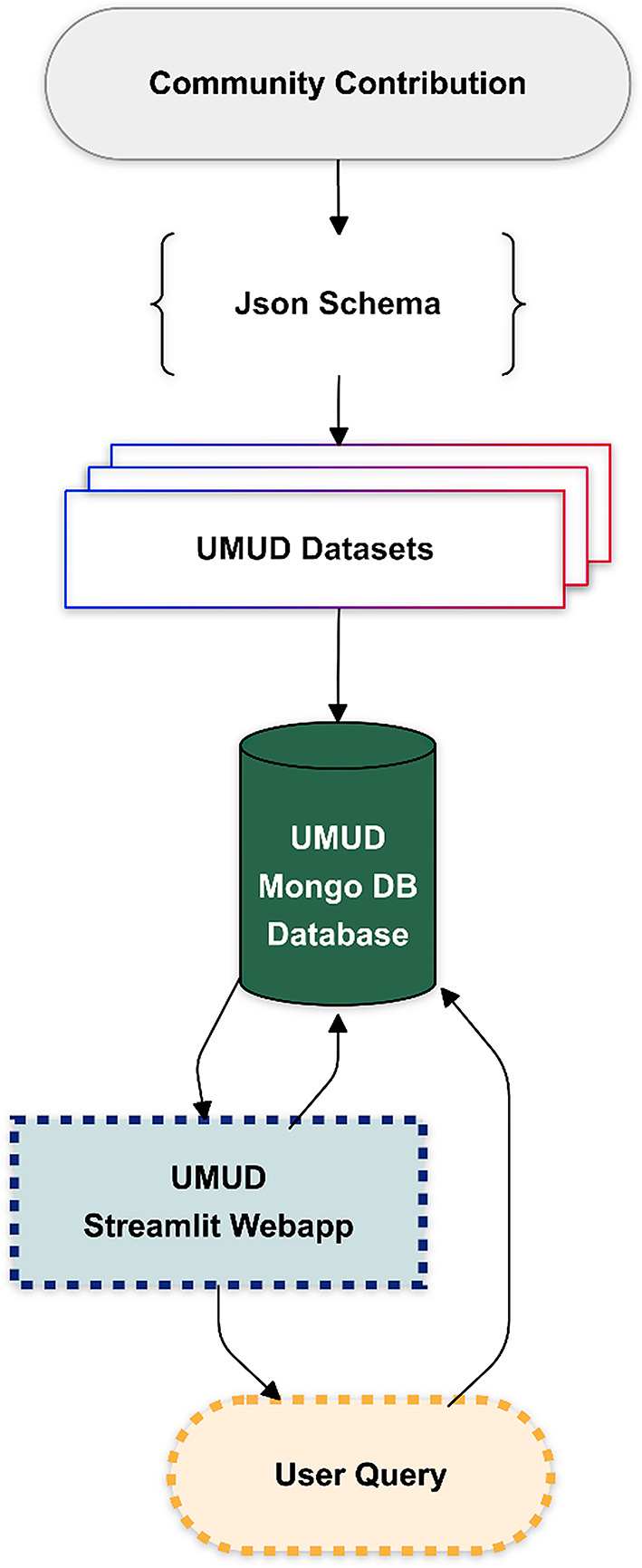
Workflow of UMUD. The UMUD database is built upon openly available datasets provided by the community. Dataset metadata is stored according to a specific JSON schema in the UMUD MongoDB database. The UMUD Streamlit web application interacts with the database based on user queries to retrieve or plot the included metadata

The UMUD repository is implemented using MongoDB Atlas (https://www.mongodb.com/) for its database infrastructure and Streamlit (v1.35.0, https://streamlit.io/) as a frontend. The UMUD code is distributed under the GNU General Public License v3.0 and hosted on Streamlit servers. The source code can be found in the corresponding GitHub repository (https://github.com/PaulRitsche/UMUD). UMUD is designed to provide seamless access to public musculoskeletal ultrasonography datasets, uploaded to trusted public repositories, such as the Open Science Framework, Zenodo, and Mendeley Data. UMUD functions like an online library, categorizing open-source datasets, but only storing their metadata and retrieving links to the datasets. It does not provide server space for images, allowing contributors the flexibility to choose where and under what terms of utilization their data is hosted, without restrictions. To facilitate easy contributions, UMUD employs Pydantic (v1.10.0) models [19] for metadata input validation and a standardized metadata JSON schema. We further provide standardized instructions for dataset creation and upload (Appendix A), according to the FAIR principles [17, 18]. This ensures consistency across datasets and minimizes the effort required for contributors to share their work. The workflow of the UMUD database is displayed in Fig. 2. The UMUD web application is designed as follows:

### Datasets

The Datasets Tab serves as the primary feature for interacting with the UMUD database. It provides a user-friendly interface for querying and retrieving datasets based on specific metadata criteria. This feature allows users to efficiently narrow down their search by applying one or more filters to customize the results. Available metadata filters include, but are not limited to, dataset name, muscle group, muscle region, ultrasound device, transducer, participants’ age, height, weight, publication DOI, and authors (see Appendix A). The content of the metadata descriptors was based on iterative selection and a final consensus among the authors. To use this feature, users simply select the desired filters, input the corresponding prompts, and execute the search query. The system then returns links to the datasets that meet the selected criteria, along with detailed descriptions.

### Database exploration tool

**Fig. 3:**
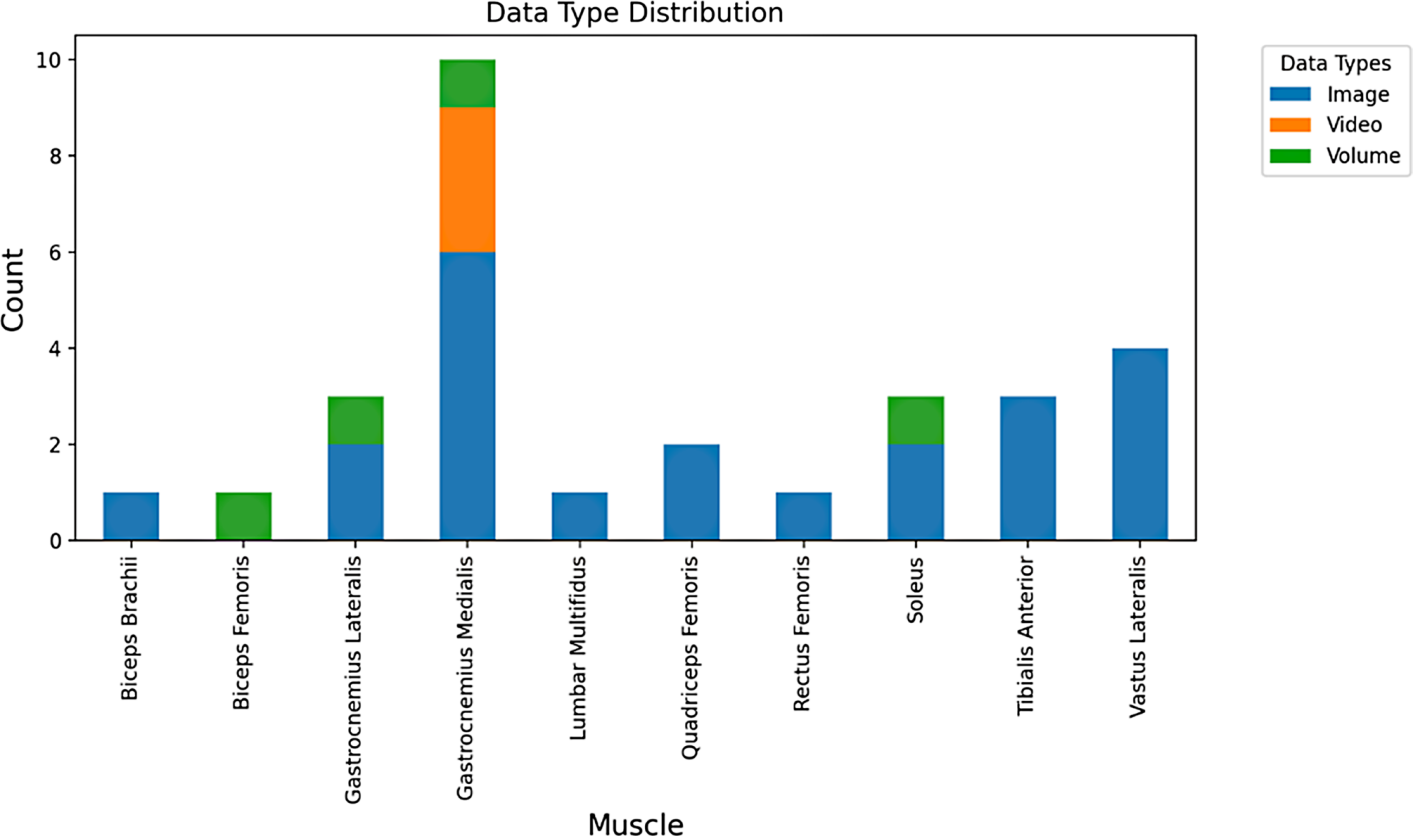
Muscle distribution and data type distribution for each muscle currently represented in the UMUD database. More plot options are available on the website and will be expanded in the future releases

The Database Exploration Tool is an alternative approach to navigate the datasets included in UMUD. The tool allows users to explore the database contents using a tabular overview and interactive charts; an example is shown in Fig. 3. Additionally, the tool facilitates users to download metadata of filtered datasets for offline analysis. This functionality allows researchers to streamline data exploration and leverage the repository’s content for their studies. So far, UMUD includes 14 datasets, which are summarized in Table 1, including 76.124 images (and/or 2.674 videos) derived from 1.901 participants. While the datasets currently included in UMUD contain only images, videos, and volume data, future datasets may also include other types of data, such as radiofrequency, elastography and power/color doppler signals, as soon as they become available.

### Benchmarks

To support operator training and comparison of automated image analysis algorithms, UMUD provides downloadable benchmark datasets. Currently, one benchmark dataset analyzed by multiple experts (i, MultExpData), one dataset containing expert-analysis-based overlays of muscle geometry (ii, ExpDrawnData), and three labeled benchmark training datasets (iii, LabTrainData) are included. (i) The MultExpData benchmark dataset includes 35 muscle architecture images and 30 panoramic cross-sectional area images from various muscles, acquired using different devices, and analyzed by six expert operators (see Appendix B for a detailed description). It will serve to test automatic analysis algorithms on a common ground truth or compare manual analysis results to an agreement of multiple experts. We will report curated comparison statistics and a ranking of available image analysis algorithms in future releases on the website. (ii) The ExpDrawnData contains 250 images with fascicles drawn by an expert operator, depicting muscle architecture from different regions of the vastus lateralis in 40 young adults (age: 25.58 ± 4.55 years) and 85 older adults (age: 75.88 ± 4.68 years) of both sexes. These annotations provide a reference for novel operators to learn and practice how to analyze fascicle length in ultrasonography images. (iii) With LabTrainData, we offer three further training datasets from previously published literature [20–22], containing labeled muscle fascicle, muscle aponeurosis, and muscle cross-sectional area images. These datasets serve as a training and validation baseline for the development of new deep-learning models to analyze muscle geometry in ultrasonography images. We will report comparison tables of the available models, calculating various performance metrics in future releases. For the same purpose, in future releases we will provide benchmark models trained on these datasets for muscle geometry analysis, developed from previously published studies [20–22].

**Table 1:** Datasets currently included in the universal musculoskeletal ultrasonography database (UMUD)

Dataset Name	Associated Publication	Year	Sample Size	Sex	Muscle	Data Type	Image Type	Data Plane
BicepsFem3DUS	Andrews et al. [23]	2024	12	Both	BF	Volume	n.a.	Longitudinal
DeepACSA	Ritsche et al. [21]	2022	153	Both	GM, GL, RF, VL	Images	Panoramic	Transverse
DeepMTJ	Leitner et al. [24]	2021	161	Both	GM, GL	Images	Static	Longitudinal
DLSegMskNeromDisease	Marzola et al. [25]	2021	1,238	Both	BB, GM, TA	Images	Static	Transverse
DL_Track_US	Ritsche et al. [22]	2023	n.a.	Both	GM, VL, SOL, TA	Images	Static	Longitudinal
FALLMUD	Cunningham et al. [26]	2017	8	Both	GM, SOL	Images	Static	Longitudinal
GasFasTrack	Drazan et al. [27]	2019	5	Both	GM	Video	Static	Longitudinal
HEELRAISE	Niemi [28]	2017	13	Both	GM	Video	Static	Longitudinal
Leg3dUS	Duque et al. [29]	2024	44	Both	GM, GL, SOL	Volume	n.a.	n.a.
LuminousMultifidus	Belasso et al. [30]	2020	109	Both	LM	Images	Static	Transverse
NeuAge_OLD	Sarto et al. [31]	2024	88	Both	QF, VL	Images	Panoramic Static	Longitudinal Transverse
NeuAge_YOUNG	Sarto et al. [31]	2024	42	Both	QF, VL	Images	Panoramic Static	Longitudinal Transverse
SimpleMuscleArchitectureAnalysis	Seynnes & Cronin [32]	2019	15	Both	GM, TA	Images	Static	Longitudinal
UltraTimTrack	van der Zee et al. [33]	2024	8	n.a.	GM	Video	Static	Longitudinal

BB: biceps brachii; BF: biceps femoris; GM: gastrocnemius medialis; GL: gastrocnemius lateralis; LM: lumbar multifidus; QF: quadriceps femoris; RF: rectus femoris; VL: Vastus lateralis; SOL: Soleus, n.a.: not applicable, panoramic: swiping motion of the probe during image acquisition and frame stitching, static: Image/Video taken without probe movement

### Image analysis

UMUD provides a curated list of state-of-the-art semi-automated and fully automated muscle geometry analysis algorithms presented in the literature. The platform currently features the following algorithms: ACSAuto [34], DeepACSA [21, 35], DeepMTJ [24], DL_Track_US [20, 22], SMA [32], TimTrack [36], UltraTimTrack [33], and UltraTrack [37]. The inclusion criteria for algorithms are open-access code and license, as well as sufficient documentation of usage. Note that almost none of the above-listed algorithms provided adequate tests to verify reproducibility. Furthermore, some of these algorithms [33, 36] are not written in an open-source language and may require additional software licenses.

## Utility and discussion

We believe that UMUD could have a significant impact on the musculoskeletal imaging community. Some of the main applications of UMUD are briefly discussed below.

### Benchmarking, transparency, and validation

UMUD could be a valuable resource for testing published and future algorithms, as its publicly available benchmark datasets allow researchers to independently analyze and validate findings from previous studies, using the same expert-based ground truth. By reporting comparison statistics, UMUD transparently reports the proficiency of available automatic analysis algorithms. Providing openly available models and benchmark data has been successful in other domains [15]. A challenge that remains is to provide detailed documentation on how to use and contribute to the benchmarks provided by UMUD, especially for users not familiar with this process [15].

### Trainee training

Ultrasound imaging is an operator-dependent technique concerning manual data analysis, and its accuracy can be influenced by the operator’s experience, particularly the variability associated with the manual digitization process [1]. By providing datasets with corresponding manual analyses from multiple expert raters, UMUD serves as a valuable resource for trainees new to manual ultrasound imaging analysis. This is in line with a recent study investigating the usage of Hugging Face [15] that showed that expert-based solutions and clarifications were the biggest benefit to the community. The inclusion of ultrasound videos represents a key strength of the dataset, as it enhances educational value by capturing the dynamic nature of sonographic assessments [38, 39]. Additionally, UMUD facilitates the adoption of semi-automated and fully automated analysis by offering a comprehensive and up-to-date list of all available algorithms proposed in the literature. As reported by Taraghi et al. [15], providing the community with problem-specific analytical solutions is needed. We believe that automated tools are crucial for advancing research and implementation in clinical practice. This is particularly important because manual analysis, though considered the gold standard, is time-consuming and subjective, especially for large datasets. Automated algorithms may overcome these challenges by delivering faster and more objective results. However, we acknowledge that the performance of the currently available algorithms is not high enough for out-of-the-box applicability. The performance and generalizability of these tools can vary significantly, depending on their development approach and the datasets used for training. While some tools are versatile and perform well across a range of ultrasonography images or videos, others may lack generalizability or be more limited in scope.

### Open data sharing

Acquiring large datasets of ultrasound scans for the assessment of skeletal muscle and tendon properties is challenging, as these scans are not typically collected for medical purposes in routine clinical practice and are governed by strict data-sharing laws. The lack of large-scale image datasets limits the development of new automated and generalizable muscle geometry analysis algorithms, as efficient implementations using deep neural networks generally require very large datasets [40]. UMUD, by facilitating access to all available datasets on musculoskeletal ultrasonography, will serve as a key resource in addressing this challenge. Furthermore, by highlighting this need, listing the advantages, and providing guidelines, UMUD might increase the incentive for open data sharing in the community [41, 42].

## Current limitations and future directions

The released v0.1.0 of UMUD still presents some limitations. First, although we provide benchmark datasets including expert analysis (MultExpData, LabTrainData), no performance metrics are available on the website yet. Secondly, due to the paucity of publicly available ultrasonography datasets, the amount of data in UMUD is relatively low compared to other platforms [43]. This also drives an imbalance in muscle representation, with the Gastrocnemius Medialis data comprising approximately 85% of the entire dataset. To overcome this limitation, we plan to continually expand UMUD by highlighting the benefits of data sharing in our community. A detailed description of how users can contribute to the project is provided in the UMUD web app. Lastly, UMUD includes only metadata and no image files. Although this allows authors maximum flexibility when hosting their data, it does not provide a centralized solution for storing musculoskeletal ultrasonography images.

The future directions of UMUD include the creation of a community challenge, improving the visualization, and expanding the included benchmarks (see Fig. 1). The community challenge will be designed to engage the community in developing models or analysis scripts to predict muscle geometrical parameters in an unseen test set of lower limb ultrasonography images. Participants will be encouraged to use any tools or techniques at their disposal to create the best predictions possible. The format of the challenge is inspired by Kaggle competitions (https://www.kaggle.com/), where participants can submit their data analysis predictions, and a leaderboard will track the top results.

## Conclusions

UMUD can help address critical challenges in data sharing and data analysis in musculoskeletal ultrasonography research. By providing a centralized repository with comprehensive metadata, benchmark datasets, and state-of-the-art algorithms, UMUD may encourage the re-use of existing data, analysis algorithm development, and comprehensive trainee training. To date, 14 datasets are included in the UMUD database, highlighting the need for community contributions. Finally, by highlighting the benefits of data sharing and offering clear guidelines, we aim to foster greater community interaction and engagement.

## Supplementary Information

Below is the link to the electronic supplementary material.

Supplementary Material 1

## Data Availability

No datasets were generated or analysed during the current study.

## Abbreviations

ORMIR: Open Repository for Musculoskeletal Imaging Research
UMUD: Universal Musculoskeletal Ultrasonography Database
