# Supplementary material for: UMUD: a web application for easy access to musculoskeletal ultrasonography datasets
Source: BMC Med Imaging. 2026 Feb 6;26:139. doi: 10.1186/s12880-026-02170-0 (PMC12998083; doi:10.1186/s12880-026-02170-0)
Supplement: Supplementary file 1 — Supplementary Material 1 [file 12880_2026_2170_MOESM1_ESM.docx]

**Supporting Information**

*Appendix A: UMUD JSON metadata descriptor file.*

{

"$schema": "http://json-schema.org/draft-07/schema#",

"title": "UMUD Dataset Metadata Schema",

"description": "A comprehensive schema for metadata of muscle ultrasound datasets in the UMUD repository.",

"type": "object",

"properties": {

"DATASET_NAME": {

"type": "string",

"pattern": "^[A-Za-z0-9]+_[0-9]{4}$",

"description": "Name of the dataset containing the name and the year separated by an underscore, e.g., 'DeepACSA_2022'."

},

"DOI": {

"type": [

"string",

"null"

],

"description": "Optional. Digital Object Identifier (DOI) of the dataset."

},

"VERSION": {

"type": "string",

"pattern": "^\\d+\\.\\d+(?:\\.\\d+)?$",

"description": "Version of the dataset following semver principles (i.e., 1.0.0)."

},

"MUSCLE": {

"type": "array",

"items": {

"type": "string"

},

"description": "List of muscles included in the dataset. Choose one or multiple from the available options."

},

"MUSCLE_REGION": {

"type": "array",

"items": {

"type": "string"

},

"description": "List of muscle regions (proximal, middle, distal). Choose one or multiple from the available options."

},

"DEVICE": {

"type": "array",

"items": {

"type": "string"

},

"description": "Optional. Ultrasound device used to collect the data. Choose one or mulitple from the available options."

},

"TRANSDUCER": {

"type": "string",

"description": "Optional. Model of the probe used during data collection. Enter one."

},

"DATA_TYPE": {

"type": "array",

"description": "Type of data in the dataset (Images, Videos, Volumes). Choose one."

},

"FILE_TYPE": {

"type": "array",

"description": "File type of the data (e.g., jpg, png, mp4). Choose one."

},

"IMAGE_TYPE": {

"type": "array",

"description": "Image type (Static, Panoramic). Choose one."

},

"IMAGE_NUMBER": {

"type": "integer",

"minimum": 0,

"description": "Number of images in the dataset."

},

"VIDEO_NUMBER": {

"type": "integer",

"minimum": 0,

"description": "Number of videos in the dataset."

},

"DATA_PLANE": {

"type": "array",

"description": "Plane in which the images/videos were collected (Transverse, Longitudinal). Choose one."

},

"SCANNING_FREQUENCY": {

"type": "integer",

"minimum": 1,

"maximum": 100,

"description": "Optional. Scanning frequency in MHz (1-100). Select value."

},

"SAMPLING_RATE": {

"type": "integer",

"minimum": 1,

"maximum": 1000,

"description": "Optional. Sampling rate or fps (0-1000). Select value."

},

"PARTICIPANT_AGE": {

"type": "number",

"minimum": 0,

"maximum": 100,

"description": "Optional. Mean age of participants (0-100). Select mean value."

},

"PARTICIPANT_HEIGHT": {

"type": "number",

"minimum": 0,

"maximum": 220,

"description": "Optional. Mean height of participants in cm (0-220). Select mean value."

},

"PARTICIPANT_WEIGHT": {

"type": "number",

"minimum": 0,

"maximum": 200,

"description": "Optional. Mean weight of participants in kg (0-200). Select mean value."

},

"PARTICIPANT_SEX": {

"type": "string",

"description": "Sex of participants (Male, Female, Both). Choose one."

},

"SAMPLE_SIZE": {

"type": "integer",

"description": "Number of participants included in the dataset (minimum 1). Enter n."

},

"DATA_LABELS": {

"type": "boolean",

"description": "Whether labels are provided for the data. Select Checkbox."

},

"DATA_LABELS_DESCRIPTION": {

"type": "string",

"maxLength": 500,

"description": "Optional. Description of the labels provided. As detailed as necessary but as short as possible."

},

"SHORT_DESCRIPTION": {

"type": "string",

"maxLength": 1000,

"description": "Brief description of the dataset (max 500 characters). As detailed as necessary but as short as possible."

},

"DATASET_YEAR": {

"type": "string",

"pattern": "^\\d{4}$",

"description": "Year the dataset was created (4-digit year)."

},

"PUBLICATION_LINK": {

"type": [

"string",

"null"

],

"pattern": "^(https?|ftp):\/\/[^\\s/$.?#].[^\\s]*$",

"description": "Optional. URL Link to the publication containing the data."

},

"DATASET_LINK": {

"type": [

"string",

"null"

],

"pattern": "^(https?|ftp):\/\/[^\\s/$.?#].[^\\s]*$",

"description": "Optional. URL Link to the publiccation containing the data."

},

"AUTHORS": {

"type": "string",

"maxLength": 500,

"description": "List of authors of the dataset, separated by commas."

},

"CONTACT": {

"type": "string",

"maxLength": 500,

"description": "List of contact emails of the authors, separated by commas."

},

"LICENSE": {

"type": "array",

"items": {

"type": "string"

},

"description": "License under which the data is shared."

},

},

"required": [

"DATASET_NAME",

"VERSION",

"MUSCLE",

"MUSCLE_REGION",

"DATA_TYPE",

"FILE_TYPE",

"IMAGE_TYPE",

"DATA_PLANE",

"PARTICIPANT_SEX",

"SAMPLE_SIZE",

"SHORT_DESCRIPTION",

"DATASET_YEAR",

"AUTHORS",

"CONTACT",

"DATASET_LINK",

"LICENSE"

]

}

*Appendix B: Multi-expert manual analysis benchmark dataset description*

## UMUD benchmark muscle architecture dataset

Version: 0.1.0

Last edited: 06.01.2024

### Analysis methodology

The images were manually analyzed using ImageJ (FIJI). For this, 3 straight lines were used to assess muscle thickness at a left, middle, and right location in the images, 3 segmented lines were used to asses 3 fascicles, and 3 angles were used to assess 3 pennation angles (not the same as for the fascicles). Of all parameters, the mean was calculated which represented the final estimate. This methodology was kept constant between all six expert raters (PR, FSar, CL, MVF, OS, and NC).

### Dataset description

The UMUD benchmark muscle architecture dataset contains 35 muscle architectural images.

Muscles included in this set are the gastrocnemius medialis, the soleus, and the vastus lateralis.

The images were acquired by four different devices: Hitachi Aloka Alpha-10, Telemed Echo Blaster 128, Philips HD11, and Telemed ArtUs EXT-1H.

The dataset contains images from both, young and healthy males and females. For examples on older individuals please see the annotated dataset.

## UMUD benchmark RF ACSA dataset

Version: 0.1.0

Last edited: 06.01.2024

### Analysis methodology

The images were manually analyzed using ImageJ (FIJI). For this, the polygon tool was selected and the area of the muscle was drawn. The inner border of the rectus femoris was followed until the whole anatomical cross-sectional area was outlined. The resulting value represented the final estimate for the image. This methodology was kept constant between all six expert raters.

### Dataset description

The UMUD benchmark RF ACSA dataset contains 30 muscle anatomical cross-sectional area images of the rectus femoris.

The images were acquired by three different devices: Siemens Acuson Juniper, Esaote MyLab 70, and Aixplorer Ultimate.

The dataset contains images from both, young and healthy males and females.
